# Cooperation and Stability through Periodic Impulses

**DOI:** 10.1371/journal.pone.0009882

**Published:** 2010-03-29

**Authors:** Bo-Yu Zhang, Ross Cressman, Yi Tao

**Affiliations:** 1 Key Laboratory of Animal Ecology and Conservation Biology, Centre for Computational Biology and Evolution, Institute of Zoology, Chinese Academy of Sciences, Beijing, People's Republic of China; 2 School of Mathematical Sciences, Beijing Normal University, Beijing, People's Republic of China; 3 Department of Mathematics, Wilfrid Laurier University, Waterloo, Ontario, Canada; University of California, Berkeley, United States of America

## Abstract

Basic games, where each individual chooses between two strategies, illustrate several issues that immediately emerge from the standard approach that applies strategic reasoning, based on rational decisions, to predict population behavior where no rationality is assumed. These include how mutual cooperation (which corresponds to the best outcome from the population perspective) can evolve when the only individually rational choice is to defect, illustrated by the Prisoner's Dilemma (PD) game, and how individuals can randomize between two strategies when neither is individually rational, illustrated by the Battle of the Sexes (BS) game that models male-female conflict over parental investment in offspring. We examine these questions from an evolutionary perspective where the evolutionary dynamics includes an impulsive effect that models sudden changes in collective population behavior. For the PD game, we show analytically that cooperation can either coexist with defection or completely take over the population, depending on the strength of the impulse. By extending these results for the PD game, we also show that males and females each evolve to a single strategy in the BS game when the impulsive effect is strong and that weak impulses stabilize the randomized strategies of this game.

## Introduction

A great deal of game-theoretic research has been devoted to explain the prevalence of cooperation in biological systems as well as in human society. One reason for the vast literature from members of the game theory community on this topic is that their methods do not work for the underlying stage game, the symmetric Prisoner's Dilemma, which pits cooperative behavior against its nemesis of defection. In particular, the only rational option in this PD stage game is to Defect since this strategy strictly dominates Cooperate (i.e. a player is better off defecting than cooperating no matter what the opponent does).

On the other hand, cooperation can be rational when the payoffs of the PD game are modified by assuming some relatedness between the players [1], [2], by them playing the game an uncertain number of times [3], or by extending the model to a multi-player (i.e. more than two) public goods game [4]. These predictions are often based on applying either static (e.g. evolutionarily stable strategy (ESS)) or dynamic (e.g. the replicator equation) methods from evolutionary game theory [5] that assumes a large population of agents paired at random to play the game. Population interactions that are structured either spatially (e.g. through nearest neighbors on a lattice) or socially (e.g. through adjacent nodes in a graph) also enhance the evolution of cooperation [6]–[11] as do the stochastic effects of finite populations [12].

To a lesser extent, the question of stability of mixed strategy equilibrium solutions (and their interpretation) has also created controversy in the game theory community [13], [14]. This is especially true of two-player non symmetric games due to the result that, at any evolutionarily stable state of such games, players must use pure strategies [15]. The controversy here is clearly demonstrated through typical payoffs used in the Battle of the Sexes game [5], [16], [17] introduced into biology by Dawkins [18] to model the conflict between males and females concerning their respective contributions to parental investment (see also the Buyer-Seller game [19] that has the same qualitative payoff structure). In the BS stage game, each player has two pure strategies and the only equilibrium solution is for both players to use a mixture of their strategies. Furthermore, the replicator equation applied to this game yields periodic solutions around this mixed strategy equilibrium pair even though Maynard Smith [17] (Chapter 11C) states that “I am unable to offer illustrative examples, or evidence that such cycles occur.”

In this article, we re-examine the PD and BS stage games from the dynamic perspective where, in addition to the continuous trajectories of evolutionary game theory, there are periodic jumps in the population size. In biological systems, these latter impulsive perturbations may be due to sudden changes in the physical environment (e.g. the effects of climate change or natural disaster) or to intrinsic diurnal/nocturnal and seasonal life history effects in the physiological and reproductive mechanisms of individuals in the population. Impulsive perturbations have also been used to model the effect on human behavior of sudden market corrections or of sudden shifts in the business cycle [20]. We assume that the impulsive “coefficient” for an individual depends only on its strategy and analyze the resultant dynamics. In particular, we give analytic conditions for the coefficients in the PD game for the successful initial invasion of Cooperators into a population of Defectors as well as conditions based on stronger impulsive effects for Cooperators to completely take over the system. We also show that these latter conditions applied to the BS game imply global convergence to a monomorphic system where all males use one pure strategy as well as all females. Moreover, when impulsive effects are weak in the BS game, a globally attracting polymorphic state emerges near the mixed equilibrium pair.

## Analysis

### Prisoner's Dilemma

The PD stage game is ubiquitous in the game theory literature and so needs no introduction. We follow the standard notation by taking its payoff matrix as
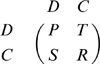
(1)where 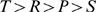. The entries in this matrix give the payoff to the row player in a two-player interaction with the column player (e.g. if a player Defects against an opponent who Cooperates, his payoff is 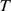). Since 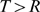 and 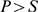, Defect strictly dominates Cooperate and so it is the only rational outcome of this one shot game.

To illustrate how periodic impulses can be combined with an evolutionary dynamics, suppose that the replicator equation (see **Eq.4a** below) models behavioral evolution. From a biological perspective, these dynamics result from a direct correspondence between expected payoff and reproductive success [21]. Specifically, if 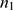 and 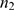 are the numbers of Defectors and Cooperators respectively in the population at time 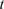, then
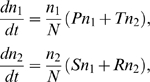
(2)where 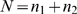 is the total population size. Here 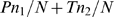 is the expected payoff to a Defector in a single interaction with a randomly chosen opponent assuming that population size is large. From Taylor and Jonker [21], the population dynamics (**Eq.2**) implies the frequency 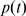 of Defectors in the population (i.e. 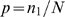) evolves according to the replicator equation (**Eq.4a**).

Now suppose that individuals die at periodic intervals 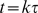 for 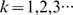. That is, reproductive success (i.e. fitness) in **Eq.2** refers only to births. Deaths cause a jump in the solution trajectories of **Eq.2** of the form 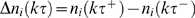 where 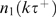 (respectively, 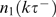) is the number of Defectors immediately after (respectively before) the jump. If the death rate of each strategy type is independent of population size, then
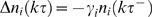
(3)for some 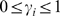. The parameter 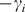 is called the “impulsive coefficient” for the *i*-th strategy. The dynamical system with periodic impulsive effects combines **Eq.2** when 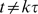 with **Eq.3** when 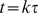. In particular, for every nonnegative initial condition 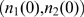, this impulsive dynamical system has a unique nonnegative solution for all 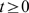.

Evolutionary game theory is more concerned with the evolution of strategy frequencies than with how the absolute numbers of strategy users evolve over time. It is shown in File S1 (see also [22]) that
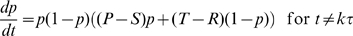
(4a)

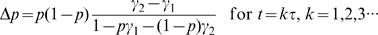
(4b)where 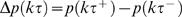 denotes the jump in 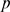 at moment 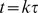. That is, 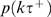 (respectively, 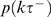) is the frequency of Defector immediately after (respectively before) the jump. For the analysis of this dynamical system, it is important to notice that the frequency dynamics **Eq.4** is independent of population size 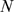. When there are no impulsive effects, we have the standard replicator equation **Eq.4a** [21]. For this reason, we call **Eq.4** the replicator equation with periodic impulses.

#### Remark

The replicator equation with periodic impulses developed above is based on continuous births throughout the season and deaths only at the end. One consequence of our assumptions is that birth and death rates are independent of population size. It can be shown [22] that dynamics **Eq.4** also emerges when birth rates are altered by any strategy-independent background fitness (which is usually assumed to decrease as population size increases). This background fitness can be used to investigate the dynamics of total population size and not only the frequency dynamics as in the standard approach to evolutionary game theory [21]. The dynamics **Eq.4** also models other periodic impulses in biological systems such as regular perturbations in the physical environment. It is well-documented [23]–[25] that humans (and other biological species) exhibit more cooperation in the face of natural disasters (also called the disaster syndrome). Such shifting of aggregate population behavior through individuals changing their strategy becomes a positive jump in the proportion of Cooperators. In fact, any impulsive coefficients in **Eq.3** satisfying 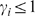 are suitable since 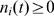 for all 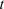 in this case. The interpretation of 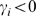 is that the impulse is then beneficial to the *i*-th strategy.

**Figure 1:**
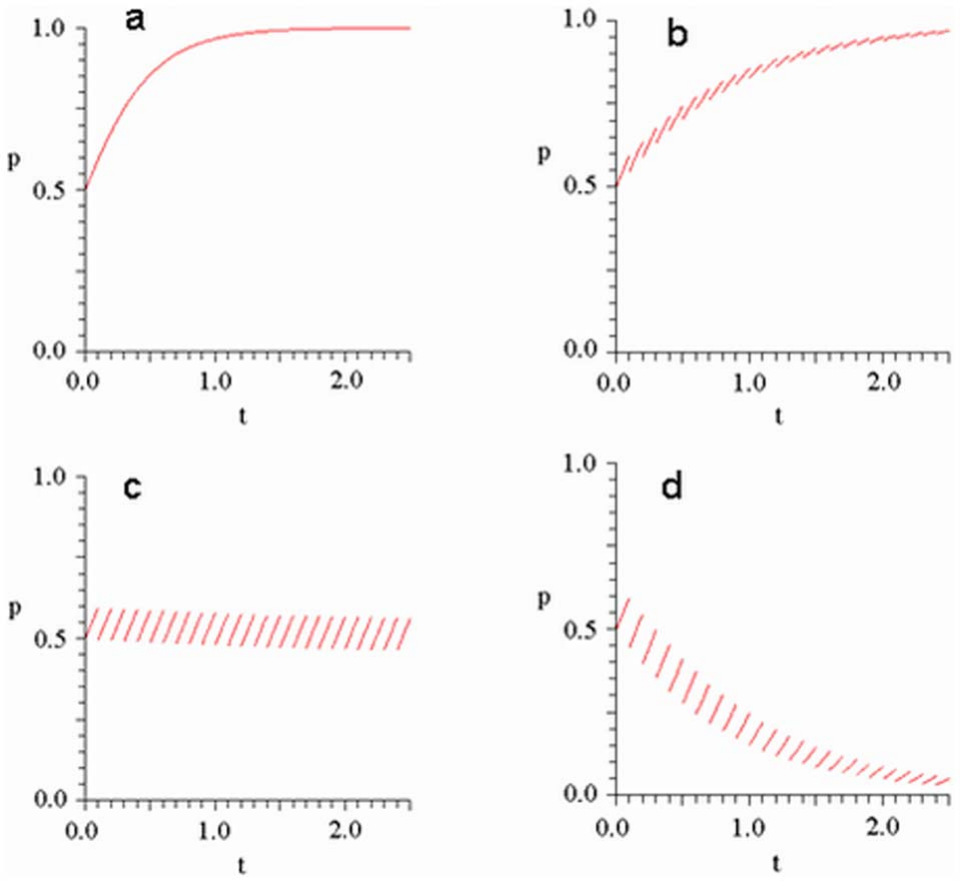
Trajectories for the replicator equation with periodic impulses (4) when payoffs 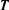, 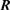, 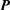, 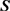 of the PD game are fixed at 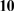, 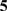, 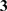, 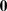 respectively and the initial frequency of Defect is 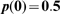. Here 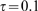 and the values taken for 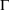 are (a) 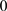; (b) 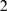; (c) 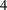; and (d) 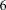.

From **Eq.4a**, 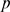 strictly increases during the season (i.e. 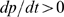) if 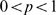 since 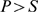 and 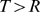. Thus, if the Defector death rate is no higher than the Cooperator (i.e. 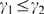), the population must evolve to all Defect since 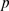 also increases at the end of each season. However, if the death rates benefit Cooperators (i.e. if 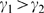), the effect of Defector deaths may offset their higher birth rates and so it is unclear which effect dominates (see Figure 1). In the extreme case where 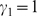, all Defectors die at the end of the first season and the population is all Cooperate thereafter.

For these reasons, we will assume that 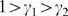 for the remainder of this section. The key to understanding the outcome in this scenario is to determine the stability of the boundary equilibria 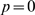 and 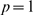 of **Eq.4**. Heuristically, when 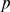 is near 0, the trajectory 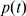 during the first season is approximated by 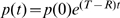 since, from **Eq.4a**, 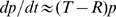. Also, from **Eq.4b**, the jump at the end of this season is 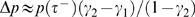. For (asymptotic) stability of all Cooperate (i.e. for 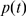 to converge to 0 if it is initially close to 0), we expect that 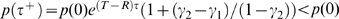. This is true if and only if
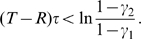
(5)Notice that 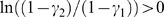 when 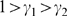 and so there are payoff matrices with 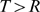 for which all Cooperate is stable.

It is proved analytically in File S1 (see also [22]) that 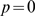 is stable if inequality **Eq.5** is true and unstable (i.e. 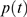 diverges from 0 if it is initially close) if this inequality is reversed (i.e. 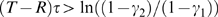). It is also shown there that all Defect is stable if
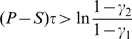
(6)and unstable if the inequality is reversed. In fact, the dynamic stability of the boundary equilibria for threshold parameters when **Eq.5** or **Eq.6** is an equality is also characterized there. Besides one exceptional case discussed in the final paragraph of this section when both **Eq.5** and **Eq.6** are equalities, there is a unique interior 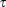-periodic trajectory (i.e. a 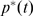 with 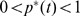 for all 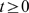 and 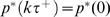 for all 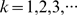) if and only if either both boundary equilibria are unstable or both are stable. In the first (respectively, second) case, 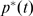 is globally stable (respectively, unstable). Finally, if exactly one boundary equilibrium is stable, then it is globally stable in that it attracts all interior trajectories.

**Figure 2:**
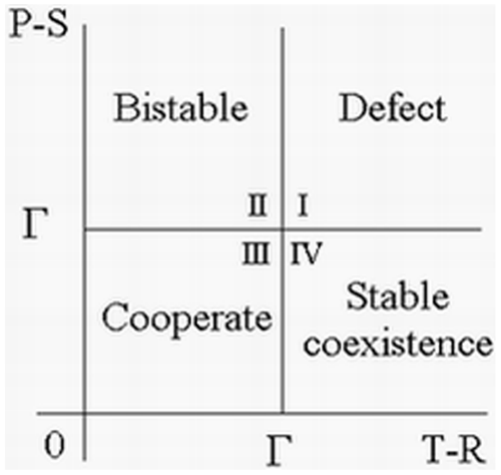
Regions of payoff parameter space determined by a fixed positive value of 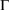 in Eq.7. In region I, 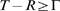 and 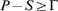, and all Defect is globally stable; in region II, 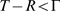 and 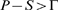, both all Cooperate and all Defect are (locally) stable, and there exists an unstable 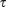-periodic solution; in region III, 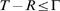 and 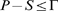, and all Cooperate is globally stable; and in region IV, 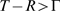 and 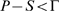, both all Cooperate and all Defect are unstable, and there exists a globally stable 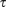-periodic solution corresponding to a mixture of Cooperators and Defectors.

These analytic results from File S1, that are summarized in the preceding paragraph, are illustrated in Figures 1 and 2 for non threshold cases. In Figure 1, trajectories of **Eq.4** are given for fixed payoff parameters and four different values of
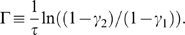
(7)In Figure 2, 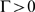 is fixed and the stability of boundary equilibria and interior 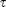-periodic trajectories is characterized in different regions of the space with parameters 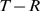 and 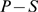.

For small values of 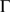, the population evolves to all Defect (Figs. 1a and 1b). An initial small population of Cooperators can successfully invade a population of Defectors once 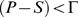. Cooperators then completely take over the population if inequality 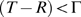 also holds (Fig. 1d and quadrant III in Fig. 2); otherwise the system approaches a globally stable 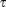-periodic state consisting of a mixture of Cooperators and Defectors (Fig. 1c and quadrant IV in Fig. 2). If 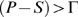 as in quadrants I and II of Figure 2, either all Defect completely takes over or we have a bistable situation where all Cooperate can persist if their initial frequency is sufficiently high.

It is instructive to consider the case of small impulsive effects (i.e. when the impulsive coefficients 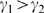 are both close to 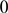). By Taylor's expansion, 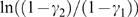 is approximately equal to 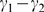. Then **Eq.7** becomes
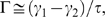
which represents the average impulsive effect over one season in favor of Cooperate. By inequalities **Eq.5** and **Eq.6**, if the positive payoff advantage during the season to Defect over Cooperate is always less than this average impulsive effect (i.e. if 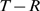 and 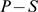 are both less than 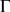), Cooperators invade and take over the population. This intuitive result can be generalized to all situations where impulsive effects are nearly equal (i.e. 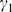 is close to 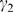 but they are not necessarily close to 0) (see also File S1). The central message here is that a small difference in the death rates of Cooperators and Defectors can have a major impact on the evolution of cooperation.

In fact, if we call 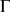 defined in **Eq.7** the average impulsive effect over one season in favor of Cooperate for any choice of 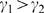, the intuitive result of the previous paragraph remains true.

In the exceptional case where 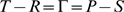, the boundary equilibria are neutrally stable and every trajectory 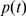 for any initial condition 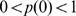 is 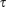-periodic. Interestingly, the special payoffs for the PD game that satisfy 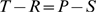 have attained prominence recently since this class includes the simplified PD games [1], [26] with payoff matrix
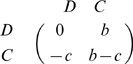
(8)Here 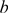 is the payoff benefit a player interacting with a Cooperator gains and 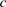 is the cost paid by Cooperators. Under the usual assumption that 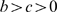, the entries in this simplified payoff matrix have the same ordering as in **Eq.1**. From **Eq.5** and **Eq.6**, if 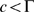 (respectively, 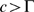), then all Cooperate (respectively, all Defect) is the final outcome. Unlike other studies on the simplified PD game [26] where the emergence of Cooperative behavior often depends on the cost-benefit ratio 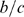, here it depends only on the cost of Cooperation. The size of the payoff benefit has no impact on our results since neither the replicator equation **Eq.4a** nor the impulse **Eq.4b** depends on 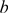. In our model, it is the impulsive benefit 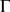 that replaces the payoff benefit 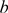. In particular, Cooperation emerges if and only if the impulsive benefit to Cooperators is greater than the cost paid by Cooperators. That is, periodic impulses that favor cooperation provide a mechanism that promotes the evolution of cooperation.

### Battle of the Sexes

In the BS stage game, male strategies are either “faithful” or “philandering” and females are “coy” or “fast” [18]. In the following two paragraphs, we briefly summarize well-known facts about this game [5], [16], [17].

If parental investment costs 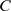, the benefit gained from an offspring is 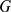 and the cost of a long engagement is 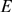, then the payoffs to males and females are given in the following bimatrix (e.g. a philanderer receives the benefit 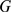 against a fast female whose net payoff is then 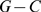).
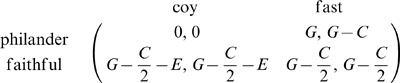
(9)With the usual assumptions that these payoffs satisfy 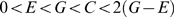, the characteristic feature of the BS game is the cyclical character of male and female best responses. If females are coy, it pays males to be faithful; if males are faithful, it pays females to be fast; if females are fast, it pays males to philander; and if males philander, it pays females to be coy. This characteristic also leads to cycling in the standard evolutionary dynamics that is concerned with the evolution of strategy frequencies.

**Figure 3:**
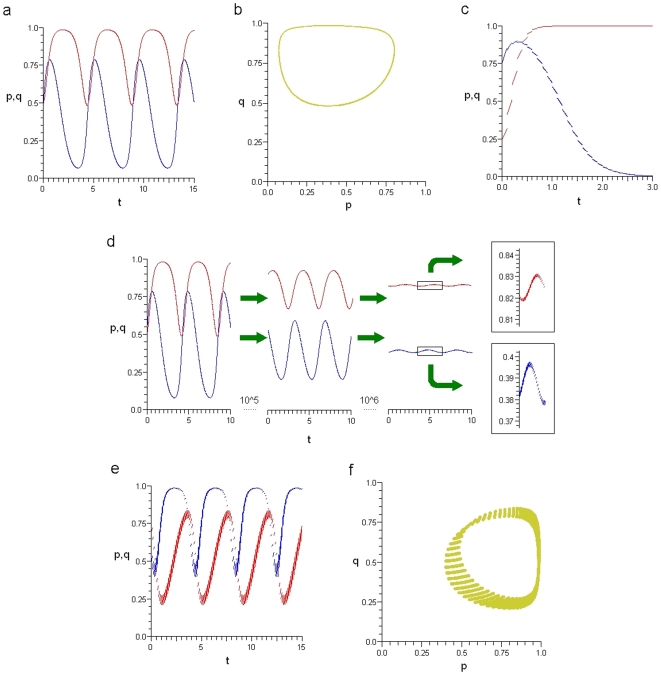
Trajectories for the bimatrix replicator equation with periodic impulses for the BS game in the 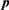-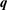 phase plane, where 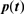 and 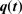 are represented by blue and red curves, respectively. The parameters are taken as 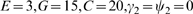 and 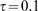, i.e., the payoff matrix is
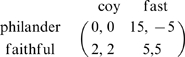
and the neutral interior equilibrium is 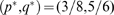. The minimum period 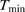 is about 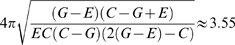. In Figure 3a–b, 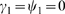 (no impulses) and the time step is from 1 to 15. All the interior trajectories are periodic orbits surrounding 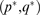. In Figure 3c, 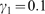 and 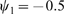 (strong impulses) and the time step is from 1 to 3. Since 
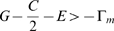
 and 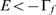, from Table 1, boundary equilibrium 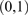 is stable and all the interior trajectories converge to 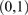. In Figure 3d, 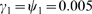 (weak impulses) and the time steps are respectively 1 to 10, 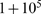 to 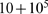 and 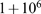 to 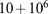. All the interior trajectories converge to an attracting set within 0.015 of the interior equilibrium 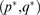. In Figure 3e–f, 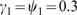 (intermediate impulses) and the time step is from 1 to 15. Clearly, interior trajectories do not always evolve to either a boundary equilibrium or to a set close to 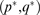.

Let 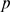 be the frequency of philanders in the male population and 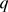 be the frequency of coy females in their population. The bimatrix replicator equation is then
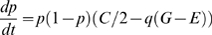
(10a)

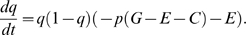
(10b)This two-dimensional dynamics on the unit square has the unique interior equilibrium 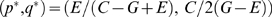 and all trajectories are periodic orbits surrounding 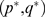 [5], [16], [17]. Figure 3a-b illustrates a typical trajectory of **Eq.10** for the payoffs.
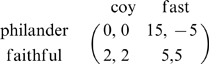
considered by Dawkins [18] that has 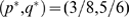.

We again assume that trajectories of the replicator equation are based on male and female births throughout the season and that there are jumps at 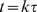 for 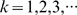 due to deaths at the end of the season. If the male (respectively, female) death rate is 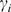 (respectively, 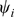) for their 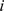-th strategy, these latter periodic impulses are
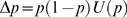
(11a)

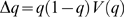
(11b)at 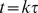 where 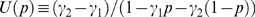 and 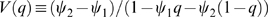. The bimatrix replicator equation with periodic impulses combines **Eq.10** at 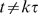 with **Eq.11** at 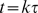.

The analysis of this impulsive dynamical system is more difficult than the replicator equation with periodic impulses for the PD game. We will assume that 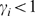 and 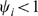 for 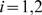. Each edge of the unit square is then invariant (as is the interior of the square). For example, on the edge where 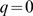, we have 
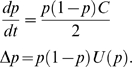
This impulsive dynamics is the same as that for the PD game with simplified payoffs **Eq.8** given by 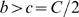.

That is, on the edge where all females are fast, the game is one where males are playing a simplified PD game among themselves. Thus, all males will eventually be faithful if they have a lower death rate than philanderers (i.e., 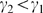) that satisfies 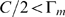 where 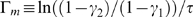 is the impulsive benefit for faithful males (cf. the discussion in the PD section about **Eq.7**). If 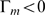, it is an impulsive benefit for philandering males. On the other hand, philanderers will take over if their death rate is lower or if 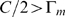. In this section, we will not consider threshold parameter values where all trajectories on an edge are 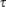-periodic. Similarly, on the edge where all males are faithful, there is a simplified PD game among females (where 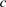 is now identified with 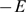). Let 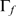 be the impulsive benefit for fast females (i.e., 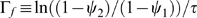). Thus, all females will eventually be fast (respectively, coy) if the cost of a long engagement is greater than (respectively, less than) the impulsive benefit for coy females, i.e. 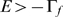) (respectively, 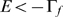).

In fact, 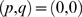 will be stable for the bimatrix replicator equation with periodic impulses (**Eq.10** and **Eq.11**) on the unit square if males are eventually faithful on the first edge and females are eventually fast on the second edge. This result is indicated in the first row of Table 1 that summaries the stability of all four vertices of the unit square. The proof is in File S1 where it is also shown that, if one vertex is stable, then it is globally stable (i.e. all trajectories in the interior of the unit square converge to it). In particular, at most one vertex can be stable.

**Table 1:** Stability of Boundary Equilibria 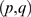 (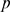 is the frequency of males who philander and 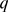 is the frequency of coy females).

Boundary Equilibrium 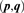	Conditions for Stability of Boundary Equilibrium
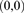	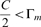 and 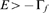
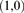	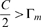 and 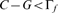
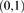	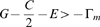 and 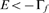
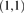	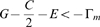 and 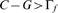

From Table 1, if impulsive effects are strong enough on any edge to reverse the flow of the replicator equation **Eq.10** there (i.e., if any of the inequalities 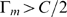, 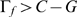, 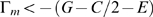, or 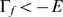 are true), then there is a globally stable pure strategy pair for males and females. In particular, these periodic impulses have removed the characteristic interior cycles of the BS game and replaced them with global stability at a vertex. Figure 3c illustrates a typical trajectory of **Eq.10**, **Eq.11** when one of the stability conditions for the vertex (1,0) in Table 1 are satisfied.

When impulses are not strong (i.e. when 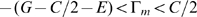 and 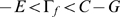), the limiting behavior of each trajectory on the boundary of the unit square is the same whether or not periodic impulses **Eq.11** are combined with the bimatrix replicator dynamics; namely, all these trajectories evolve to the first vertex encountered in a counterclockwise direction. On the other hand, no interior trajectory evolves to a point on the boundary. Figure 3e–f illustrates a typical trajectory when impulses are of intermediate strength. Notice that this trajectory does not surround 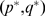 since the impulses occur before it has enough time to do so. In fact, through simulations (see File S1), it is apparent that the properties of such trajectories are quite complex with multiple 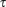-periodic solutions possible. The number 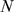 of these solutions appears to depend linearly on the ratio of 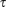 to the minimum period 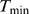 of interior periodic cycles of the bimatrix replicator dynamics **Eq.10** in that it is approximated by 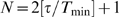 where [ ] is the integer part of a positive real number. Proofs of these conjectures suggested by simulations are beyond the current techniques available to analyze these impulsive systems. We are hopeful that game-theoretic intuition will extend these techniques in future research and, in the process, improve our understanding of how arbitrary periodic impulses affect the evolutionary dynamics, especially for non symmetric two-player games (such as the BS game) with a unique equilibrium in the interior of the strategy space.

Finally, for weak impulses (i.e. for 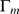 and 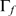 both close to 0 and at least one nonzero) with 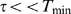, simulations show that all interior trajectories evolve to a small neighborhood of 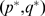 (see Figure 3d). Specifically, although the limiting behavior is not exactly the interior equilibrium, the large periodic cycles of the bimatrix replicator dynamics are replaced by orbits that become arbitrarily close to this equilibrium as the impulsive effect weakens. In fact, except for the one 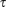-periodic solution, it appears that all trajectories approach the single orbit in Figure 3d (which is not 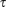-periodic).

In summary, both strong and weak impulsive effects promote stability in BS games. Strong impulses imply the system has a globally stable outcome where all males adopt the same strategy as do all females (i.e. both sexes exhibit monomorphic behavior). On the other hand, weak impulses eliminate the wild fluctuations typical of the periodic cycles of the bimatrix replicator dynamics **Eq.10** and replace them with an attracting set near its interior equilibrium that consists of a stable polymorphic population of males and females.

## Results and Discussion

Our model combining periodic impulses with an evolutionary dynamics is based on several simplifying assumptions. First, population sizes are assumed to be sufficiently large that stochastic effects due to finite populations are ignored in the deterministic dynamics and fitness is given by expected payoff as in the original development of the replicator equation [21]. Our assumption that death rates depend only on strategy type then implies total population size does not influence the evolution of strategy frequencies given by our replicator equation with periodic impulses. Moreover, in the BS game, we have adopted the unstated common assumption of evolutionary game theory applied to asymmetric games with a bimatrix payoff matrix [5] that individual fitness is based on one random interaction per unit time between different types of individuals.

The analysis of the effects of periodic impulses becomes more complex when finite populations and/or unequal population sizes of different types in asymmetric games are included. In particular, the dynamics of total population size must then be taken into account. From this perspective, our analysis of the frequency effects of periodic impulses can be viewed as a base model against which these more complex systems can be compared, in much the same way that the replicator equation of evolutionary game theory has served as a means to gain an intuitive understanding of behavioral evolution. Our analytic results, that characterize when periodic impulses favoring cooperation in the PD game can overcome the selective advantage of defection and when both strong and weak impulses have a stabilizing effect in the BS game, can then be tested (perhaps numerically) to see if they continue to hold in more complicated models that do not satisfy our simplifying assumptions.

## Supporting Information

File S1 Supporting information for “Stability and Cooperation through Periodic Impulses”.
(1.03 MB DOC)
