# Supplementary material for: Cooperation and Stability through Periodic Impulses
Source: PLoS One. 2010 Mar 29;5(3):e9882. doi: 10.1371/journal.pone.0009882 (PMC2848027; doi:10.1371/journal.pone.0009882)
Supplement: File S1 — Supporting information for “Stability and Cooperation through Periodic Impulses”. (1.03 MB DOC) [file pone.0009882.s001.doc]

**Cooperation and Stability through Periodic Impulses**

***Supporting Information***

Bo-Yu Zhang, Ross Cressman and Yi Tao

**I. PD Game**

For the PD game, the evolution of the numbers of Defectors and Cooperators, denoted respectively by $n_{1}$ and $n_{2}$, is

$\begin{matrix} \frac{dn_{1}}{dt}=\frac{n_{1}}{N}\left(Pn_{1}+Tn_{2}\right) \\ \frac{dn_{2}}{dt}=\frac{n_{2}}{N}\left(Sn_{1}+Rn_{2}\right) \end{matrix}$ (S. 1)

for $t\neq k\tau$ ($k=1,2,3,\cdot\cdot\cdot$). When $t=k\tau$ ($k=1,2,3,\cdot\cdot\cdot$), the jumps in $n_{1}$ and $n_{2}$ at $t=k\tau$ ($k=1,2,3,\cdot\cdot\cdot$) are given by

$\begin{matrix} \Delta n_{1}\left(k\tau\right)=-\gamma_{1}n_{1}\left(k\tau^{-}\right) \\ \Delta n_{2}\left(k\tau\right)=-\gamma_{2}n_{2}\left(k\tau^{-}\right) \end{matrix}$ (S. 2)

where $\Delta n_{i}\left(k\tau\right)$ is defined as $\Delta n_{i}\left(k\tau\right)\equiv n_{i}\left(k\tau^{+}\right)-n_{i}\left(k\tau^{-}\right)$. With$p\equiv n_{1}/N$, the replicator equation for the time evolution of $p$ is

$\frac{dp}{dt}=p\left(1-p\right)\left(\left(P-S\right)p+\left(T-R\right)\left(1-p\right)\right)$ (S. 3a)

for $t\neq k\tau$. Similarly, the jump in $p$ at $t=k\tau$ ($k=1,2,3,\cdot\cdot\cdot$) is given by

$$\begin{matrix} \Delta p\left(k\tau\right)\equiv p\left(k\tau^{+}\right)-p\left(k\tau^{-}\right) \\ \text{ }=\frac{n_{1}\left(k\tau^{+}\right)}{n_{1}\left(k\tau^{+}\right)+n_{2}\left(k\tau^{+}\right)}-p\left(k\tau^{-}\right) \\ \text{ }=\frac{\left(1-\gamma_{1}\right)p\left(k\tau^{-}\right)}{\left(1-\gamma_{1}\right)p\left(k\tau^{-}\right)+\left(1-\gamma_{2}\right)\left(1-p\left(k\tau^{-}\right)\right)}-p\left(k\tau^{-}\right) \\ \text{ }=p\left(k\tau^{-}\right)\left(1-p\left(k\tau^{-}\right)\right)\frac{\gamma_{2}-\gamma_{1}}{1-\gamma_{1}p\left(k\tau^{-}\right)-\gamma_{2}\left(1-p\left(k\tau^{-}\right)\right)}\text{ .} \end{matrix}$$

That is, we have

$\Delta p=p\left(1-p\right)\frac{\gamma_{2}-\gamma_{1}}{1-\gamma_{1}p-\gamma_{2}\left(1-p\right)}$ (S. 3b)

for $t=k\tau$ ($k=1,2,3,\cdot\cdot\cdot$). Combining **Eqs.** **S.3a** and **S.3b**, we get the replicator equation with periodic impulses

$\frac{dp}{dt}=p\left(1-p\right)\left(a_{2}-p\left(a_{1}+a_{2}\right)\right)\text{ }t\neq k\tau$ (S.4a)

$\Delta p=p\left(1-p\right)U\left(p\right)\text{ }t=k\tau\text{ for }k=1,2,3,\cdot\cdot\cdot$ (S.4b)

where $a_{1}\equiv S-P<0$, $a_{2}\equiv T-R>0$ and $U\left(p\right)\equiv\left(\gamma_{2}-\gamma_{1}\right)/\left(1-\gamma_{1}p-\gamma_{2}\left(1-p\right)\right)<0$ when $\gamma_{2}<\gamma_{1}<1$. This system was analyzed for two-strategy symmetric games by Wang et al. [1]. The following analysis uses a different approach that rests on translating the system to a difference equation (**Eq.** **S.5**). The generalization of this difference equation method to the BS game is needed to prove the existence of an interior $\tau$-periodic solution in this game (see Theorem 2 below).

Clearly, for **Eq.** **S.4** both $p=0$ and $p=1$ are boundary equilibria. In order to determine the existence of the interior $\tau$-periodic solution of (S.4), let $F\left(p\right)\equiv p\left((k+1)\tau^{-}\right)-p\left(k\tau^{-}\right)$. Then $p^{*}\left(t\right)$ is an interior $\tau$-periodic solution of (S. 4) if and only if $F\left(p^{*}\right)=0$. That is, $p^{*}\equiv p^{*}\left(k\tau^{-}\right)=p^{*}\left((k+1)\tau^{-}\right)$ since

$p\left((k+1)\tau^{-}\right)=p\left(k\tau^{+}\right)+\int_{k\tau}^{(k+1)\tau} G\left(p\left(t\right)\right)\text{ }dt$ ,

where $G\left(p\right)\equiv p\left(1-p\right)\left(a_{2}-p\left(a_{1}+a_{2}\right)\right)$ and $p\left(t\right)$ is the solution to (S. 4a) for $k\tau\leq t\leq(k+1)\tau$ with initial condition $p\left(k\tau^{+}\right)=p\left(k\tau^{-}\right)+\Delta p\left(k\tau\right)$.

From the definition of $F\left(p\right)$, we have also

$F\left(p\right)=\Delta p+\int_{k\tau}^{(k+1)\tau} G\left(p\right)\text{ }dt$.

The boundary equilibrium $p=0$ (or $p=1$), or an interior $\tau$-periodic solution, is stable if and only if it is a stable equilibrium of the difference equation

$p\left((k+1)\tau^{-}\right)=p\left(k\tau^{-}\right)+F\left(p\right)$ , (S. 5)

where $F\left(p\right)$ is continuous for $p\in\left[0,1\right]$, and $F\left(0\right)=F\left(1\right)=0$.

1. **Case** $a_{1}+a_{2}=0$

Notice that $G\left(p\right)=a_{2}p\left(1-p\right)$. Thus, for $k\tau\leq t\leq(k+1)\tau$ the solution of (S.4a), $p\left(t\right)$, can be expressed as $p\left(t\right)=Ce^{a_{2}t}/\left(1+Ce^{a_{2}t}\right)$ with $C=p_{0}/\left(1-p_{0}\right)$ where $p_{0}$ denotes the initial value. This implies that

$F\left(p\right)=\frac{Ce^{a_{2}\tau}}{1+Ce^{a_{2}\tau}}-p$ ,

where $C=\left(p+\Delta p\right)/\left(1-\left(p+\Delta p\right)\right)$. It is straightforward to show that $F\left(p\right)=0$ for all possible $p$ in the interval $0<p<1$ if $a_{2}\tau+\ln\left(\left(1-\gamma_{1}\right)/\left(1-\gamma_{2}\right)\right)=0$. On the other hand, if $a_{2}\tau+\ln\left(\left(1-\gamma_{1}\right)/\left(1-\gamma_{2}\right)\right)\neq0$, then no solution of equation $F\left(p\right)=0$ can exist. This implies that under the condition $a_{1}+a_{2}=0$, if $a_{2}\tau+\ln\left(\left(1-\gamma_{1}\right)/\left(1-\gamma_{2}\right)\right)=0$, then for any initial value in the interval $0<p_{0}<1$, $p\left(t,p_{0}\right)$ will be a $\tau$-periodic solution of (S.4), and it is neutrally stable (see also [1]). Conversely, if $a_{2}\tau+\ln\left(\left(1-\gamma_{1}\right)/\left(1-\gamma_{2}\right)\right)\neq0$ under the condition $a_{1}+a_{2}=0$, then no interior $\tau$-periodic solution can exist. In fact, since $Ce^{a_{2}\tau}/\left(1+Ce^{a_{2}\tau}\right)$ is an increasing function of $a_{2}\tau$, if $a_{2}\tau+\ln\left(\left(1-\gamma_{1}\right)/\left(1-\gamma_{2}\right)\right)<0$, then $F\left(p\right)<0$ for all $0<p<1$, i.e., $p=0$ is globally stable. Similarly, if $a_{2}\tau+\ln\left(\left(1-\gamma_{1}\right)/\left(1-\gamma_{2}\right)\right)>0$, then $F\left(p\right)>0$ for all $0<p<1$, i.e., $p=1$ is globally stable.

**2. Stability of boundary equilibria under** $a_{1}+a_{2}\neq0$

Define $\Phi\left(p\right)\equiv\int_{p+\Delta p}^{p} ds/G\left(s\right)$, i.e., $\Phi\left(p\right)$ is the amount of time for the change of the Defector frequency from $p+\Delta p$ to $p$. Notice that

$$\begin{matrix} \Phi\left(p\right)=\int_{p+\Delta p}^{p} \frac{1}{G\left(s\right)}\text{ }ds=\int_{p+\Delta p}^{p} \left[\frac{1}{a_{2}s}-\frac{1}{a_{1}\left(1-s\right)}+\frac{\left(a_{1}+a_{2}\right)^{2}}{a_{1}a_{2}\left(a_{2}-s\left(a_{1}+a_{2}\right)\right)}\right] \\ \text{ }=\frac{1}{a_{2}}\ln\frac{p}{p+\Delta p}+\frac{1}{a_{1}}\ln\frac{1-p}{1-\left(p+\Delta p\right)}-\frac{a_{1}+a_{2}}{a_{1}a_{2}}\ln\frac{a_{2}-p\left(a_{1}+a_{2}\right)}{a_{2}-\left(p+\Delta p\right)\left(a_{1}+a_{2}\right)} \\ \text{ }=\frac{1}{a_{2}}\ln\frac{1}{1+\left(1-p\right)U\left(p\right)}+\frac{1}{a_{1}}\ln\frac{1}{1-pU\left(p\right)}-\frac{a_{1}+a_{2}}{a_{1}a_{2}}\ln\frac{a_{2}-p\left(a_{1}+a_{2}\right)}{a_{2}-\left(p+\Delta p\right)\left(a_{1}+a_{2}\right)}\text{ ,} \end{matrix}$$

and $\lim_{p\to0^{+}} \Phi\left(p\right)=\left(1/a_{2}\right)\ln\left(\left(1-\gamma_{2}\right)/\left(1-\gamma_{1}\right)\right)$. Thus, the boundary $p=0$ is stable if $\left(1/a_{2}\right)\ln\left(\left(1-\gamma_{2}\right)/\left(1-\gamma_{1}\right)\right)>\tau$ ($a_{2}\tau+\ln\left(\left(1-\gamma_{1}\right)/\left(1-\gamma_{2}\right)\right)<0$), and it is unstable if $a_{2}\tau+\ln\left(\left(1-\gamma_{1}\right)/\left(1-\gamma_{2}\right)\right)>0$. For the situation with $a_{2}\tau+\ln\left(\left(1-\gamma_{2}\right)/\left(1-\gamma_{1}\right)\right)=0$ (i.e., $\lim_{p\to0^{+}} \Phi\left(p\right)=\tau$), notice that

$$\frac{d\Phi\left(p\right)}{dp}=-\frac{\left(a_{1}+a_{2}\right)U\left(p\right)}{\left(a_{2}-p\left(a_{1}+a_{2}\right)\right)\left(a_{2}-\left(p+\Delta p\right)\left(a_{1}+a_{2}\right)\right)}\text{ }$$

and $\lim_{p\to0^{+}} d\Phi\left(p\right)/dp=-\left(a_{1}+a_{2}\right)\left(\gamma_{2}-\gamma_{1}\right)/\left(a_{2}^{2}\left(1-\gamma_{2}\right)\right)$. Thus, when $p$ is sufficiently close to $0$, $\Phi\left(p\right)>\tau$ if $a_{1}+a_{2}<0$, i.e., $p=0$ is stable; and $\Phi\left(p\right)<\tau$ if $a_{1}+a_{2}>0$, i.e., $p=0$ is unstable.

For convenience, define

$\Gamma\equiv\frac{1}{\tau}\ln\left(\left(1-\gamma_{2}\right)/\left(1-\gamma_{1}\right)\right)$.

The stability of boundary equilibria is given by the following theorem and summarized in Table S1. The above analysis of Cases 1 and 2 provides the proofs for **(a)**, **(b)** and **(e)** in Theorem 1. The proofs of **(c)** and **(d)** are similar**.**

**Theorem 1.** *For the stability of the boundary $p=0$,* ***(a)*** *if $a_{1}+a_{2}<0$, then $p=0$ is stable if and only if $a_{2}<\Gamma$ and unstable if and only if $a_{2}\geq\Gamma$;* ***(b)*** *if $a_{1}+a_{2}>0$, then $p=0$ is stable if and only if $a_{2}\leq\Gamma$ and unstable if and only if $a_{2}>\Gamma$. Similarly, for the stability of the boundary $p=1$,* ***(c)*** *if $a_{1}+a_{2}<0$, then $p=1$ is stable if and only if $a_{1}+\Gamma<0$ and unstable if and only if $a_{1}+\Gamma\geq0$;* ***(d)*** *if $a_{1}+a_{2}>0$, then $p=1$ is stable if and only if $a_{1}+\Gamma\leq0$ and unstable if and only if $a_{1}+\Gamma>0$.* ***(e)*** *As a special case with $a_{1}+a_{2}=0$, if $a_{2}=\Gamma$ (i.e. $a_{1}+\Gamma=0$), then for any initial value in the interval $0<p_{0}<1$, $p\left(t,p_{0}\right)$ will be a $\tau$-periodic solution; and if $a_{2}\neq\Gamma$ (i.e. $a_{1}+\Gamma\neq0$), then no interior periodic solution can exist. In this situation, if $a_{2}<\Gamma$ (i.e. $a_{1}+\Gamma>0$), $p=0$ is globally stable; and if $a_{2}>\Gamma$ (i.e. $a_{1}+\Gamma<0$), $p=1$ is globally stable.*

**Table S1**

| Boundaries | | Stable | Unstable | Neutral |
| --- | --- | --- | --- | --- |
| $p=0$ | $a_{1}+a_{2}<0$ | $a_{2}<\Gamma$ | $a_{2}\geq\Gamma$ | —— |
|  | $a_{1}+a_{2}=0$ | $a_{2}<\Gamma$ | $a_{2}>\Gamma$ | $a_{2}=\Gamma$ |
|  | $a_{1}+a_{2}>0$ | $a_{2}\leq\Gamma$ | $a_{2}>\Gamma$ | —— |
| $p=1$ | $a_{1}+a_{2}<0$ | $a_{1}+\Gamma<0$ | $a_{1}+\Gamma\geq0$ | —— |
|  | $a_{1}+a_{2}=0$ | $a_{1}+\Gamma<0$ | $a_{1}+\Gamma>0$ | $a_{1}+\Gamma=0$ |
|  | $a_{1}+a_{2}>0$ | $a_{1}+\Gamma\leq0$ | $a_{1}+\Gamma>0$ | —— |

1. **Existence and stability of interior $\tau$-periodic solution under** $a_{1}+a_{2}\neq0$

We first show that there exists at most one interior $\tau$-periodic solution of **Eq.** **S.4**. Suppose that $p^{*}\left(t\right)$ is an interior $\tau$-periodic solution of (S.4), i.e. $p^{*}\equiv p^{*}\left(k\tau^{-}\right)=p^{*}\left((k+1)\tau^{-}\right)$ and $F\left(p^{*}\right)=0$). At $p=p^{*}$ we have that

$$\begin{matrix} \frac{dF\left(p\right)}{dp}=\frac{d\Delta p}{dp}+\frac{d}{dt}\left(\int_{k\tau}^{(k+1)\tau} G\left(p\right)\text{ }dt\right)\frac{dt}{dp} \\ \text{ }=\frac{\left(1-2p^{*}\right)\Delta p^{*}-\left(\Delta p^{*}\right)^{2}}{p^{*}\left(1-p^{*}\right)}+\frac{G\left(p^{*}\right)-G\left(p+\Delta p^{*}\right)}{G\left(p^{*}\right)} \\ \text{ }=\frac{\left(a_{2}-p^{*}\left(a_{1}+a_{2}\right)\right)\left(\left(1-2p^{*}\right)\Delta p^{*}-\left(\Delta p^{*}\right)^{2}\right)+G\left(p^{*}\right)-G\left(p+\Delta p^{*}\right)}{G\left(p^{*}\right)} \\ \text{ }=\frac{\Delta p^{*}\left(a_{1}+a_{2}\right)}{G\left(p^{*}\right)}\left(p^{*}\left(1-p^{*}\right)+\Delta p^{*}\left(1-2p^{*}-\Delta p^{*}\right)\right) \end{matrix}$$

since

$$\begin{matrix} G\left(p\right)-G\left(p+\Delta p\right)=p\left(1-p\right)\left(a_{2}-p\left(a_{1}+a_{2}\right)\right) \\ \text{ }-\left(p+\Delta p\right)\left(1-p-\Delta p\right)\left(a_{2}-\left(p+\Delta p\right)\left(a_{1}+a_{2}\right)\right) \\ \text{ }=\Delta p\left(a_{1}+a_{2}\right)\left(p\left(1-p\right)+\Delta p\left(1-2p-\Delta p\right)\right) \\ \text{ }-\left(a_{2}-p\left(a_{1}+a_{2}\right)\right)\Delta p\left(1-2p-\Delta p\right)\text{ .} \end{matrix}$$

Notice that $\Delta p^{*}<0$, $G\left(p^{*}\right)>0$ and

$$\begin{matrix} \left(p^{*}\left(1-p^{*}\right)+\Delta p^{*}\left(1-2p^{*}-\Delta p^{*}\right)\right)=p^{*}\left(1-p^{*}\right)\frac{\left(1-\gamma_{1}\right)\left(1-\gamma_{2}\right)}{1-\gamma_{1}p^{*}-\gamma_{2}\left(1-p^{*}\right)} \\ \text{ }=p^{*}\left(1-p^{*}\right)\left(1+\left(1-p^{*}\right)U\left(p^{*}\right)\right)\left(1-p^{*}U\left(p^{*}\right)\right)>0\text{ .} \end{matrix}$$

Thus $\left.dF\left(p\right)/dp\right|_{p=p^{*}}$ is positive if $a_{1}+a_{2}<0$ and negative if $a_{1}+a_{2}>0$. In particular, $\left.dF\left(p\right)/dp\right|_{p=p^{*}}$ is non zero. Since $\left.dF\left(p\right)/dp\right|_{p=p^{*}}$ has the same sign for all possible $p^{*}$ in the interval $0<p^{*}<1$, there exists at most one $p^{*}$. That is, if an interior $\tau$-periodic solution exists, then it is unique.

Clearly, from the boundary stability, no interior $\tau$-periodic solution can exist unless both $p=0$ and $p=1$ are stable or both are unstable. If $p=0$ is stable (unstable), then $F\left(p\right)<0$ ($F\left(p\right)>0$) for $p$ near $0$; and if $p=1$ is stable (unstable), then $F\left(p\right)>0$ ($F\left(p\right)<0$) for $p$ near $1$. From the continuity of $F\left(p\right)$, there is at least one $p$ in the interval $0<p<1$ such that $F\left(p\right)=0$. Thus, only one $p^{*}$ can exist under the condition $a_{1}+a_{2}\neq0$. That is, if both $p=0$ and $p=1$ are stable or unstable, then (S.4) has a unique interior $\tau$-periodic solution.

Notice that $p\left((k+1)\tau^{-}\right)=p\left(k\tau^{-}\right)+F\left(p\right)$ (see **Eq.** **S.5**). Thus, $p^{*}$ is an asymptotically stable equilibrium of (S.5) if and only if $-1<\left.dF\left(p\right)/dp\right|_{p=p^{*}}<0$, or $p^{*}\left(t\right)$ is a stable interior $\tau$-periodic solution of (S.4) if $-1<\left.dF\left(p\right)/dp\right|_{p=p^{*}}<0$. It is easy to see that if $p^{*}$ is locally asymptotically stable, then it must also be globally asymptotically stable.

For stability of the interior$\tau$-periodic solution we have that: **(i)** If both boundaries $p=0$ and $p=1$ are stable, or unstable, then there must exist a unique $p^{*}$ with $0<p^{*}<1$ such that $F\left(p^{*}\right)=0$; **(ii)** If one boundary is stable but the other unstable, then no interior $\tau$-periodic solution can exist; **(iii)** If both boundaries are stable, then there is an interior$\tau$-periodic solution but it is unstable since $\left.dF\left(p\right)/dp\right|_{p=p^{*}}>0$; and (iv) if both boundaries are unstable, then there is an interior$\tau$-periodic solution and it is globally asymptotically stable since $\left.-1<dF\left(p\right)/dp\right|_{p=p^{*}}<0$.

1. **BS Game**

For the BS game, let $n_{1}$ and $n_{2}$ be the numbers of philandering and faithful individuals in the male population, respectively, and $m_{1}$ and $m_{2}$ be the numbers of coy and fast individuals in the female population, respectively. Then we have

$\begin{matrix} \frac{dn_{1}}{dt}=n_{1}G(1-q), \\ \frac{dn_{2}}{dt}=n_{2}((G-\frac{C}{2}-E)q+(G-\frac{C}{2})(1-q)), \\ \frac{dm_{1}}{dt}=m_{1}(G-\frac{C}{2}-E)(1-p), \\ \frac{dm_{2}}{dt}=m_{2}((G-C)p+(G-\frac{C}{2})(1-p)), \end{matrix}$ (S.6)

where $p=n_{1}/(n_{1}+n_{2})$ is the frequency of philanders in the male population and $q=m_{1}/(m_{1}+m_{2})$ is the frequency of coy females in their population. At time $t=k\tau$ ($k=1,2,\cdot\cdot\cdot$), the jumps in $n_{i}$ and $m_{i}$ ($i=1,2$) are $\Delta n_{i}=-\gamma_{i}n_{i}$ and $\Delta m_{i}=-\psi_{i}m_{i}$, respectively.

The bimatrix replicator equation is

$\begin{matrix} \frac{dp}{dt}=p\left(1-p\right)\left(C/2-q\left(G-E\right)\right), \\ \frac{dq}{dt}=q\left(1-q\right)\left(-p\left(G-E-C\right)-E\right) \end{matrix}$ (S.7a)

for $t\neq k\tau$ ($k=1,2,\cdot\cdot\cdot$). Similar to the PD game model, the jumps in $p$ and $q$ at time $t=k\tau$ ($k=1,2,\cdot\cdot\cdot$) are given by

$\begin{matrix} \Delta p=p\left(1-p\right)\frac{\gamma_{2}-\gamma_{1}}{1-\gamma_{1}p-\gamma_{2}(1-p)} \\ \Delta q=q\left(1-q\right)\frac{\psi_{2}-\psi_{1}}{1-\psi_{1}q-\psi_{2}(1-q)}\text{ .} \end{matrix}$ (S.7b)

Combining **Eqs.** **S.7a** and **S.7b** together, we have that

$\frac{dp}{dt}=p\left(1-p\right)\left(a_{2}-q\left(a_{1}+a_{2}\right)\right)\text{ for }t\neq k\tau$ (S. 8a)

$\frac{dq}{dt}=q\left(1-q\right)\left(b_{2}-p\left(b_{1}+b_{2}\right)\right)\text{ for }t\neq k\tau$ (S. 8b)

$\Delta p=p\left(1-p\right)U\left(p\right)\text{ for }t=k\tau,\text{ }k=1,2,3,\cdot\cdot\cdot$ (S. 8c)

$\Delta q=q\left(1-q\right)V\left(q\right)\text{ for }t=k\tau,\text{ }k=1,2,3,\cdot\cdot\cdot$ (S. 8d)

where **(i)** $a_{1}=G-C/2-E>0$, $a_{2}=C/2>0$, $b_{1}=G-C<0$ and $b_{2}=-E<0$; **(ii)** $\Delta p\left(k\tau\right)=p\left(k\tau^{+}\right)-p\left(k\tau^{-}\right)$ and $\Delta q\left(k\tau\right)=q\left(k\tau^{+}\right)-q\left(k\tau^{-}\right)$ denotes the jump in $p$and $q$ at moment $t=k\tau$, respectively; and **(iii)** $U(p)$ and $V(q)$ are given by

$$\begin{matrix} U\left(p\right)=\frac{\gamma_{2}-\gamma_{1}}{1-\gamma_{1}p-\gamma_{2}\left(1-p\right)}\text{ ,} \\ V\left(q\right)=\frac{\psi_{2}-\psi_{1}}{1-\psi_{1}q-\psi_{2}\left(1-q\right)}\text{ .} \end{matrix}$$

Clearly, **Eq.** **S.8** can be equivalently expressed as the difference equations

$p\left((k+1)\tau^{-}\right)=p\left(k\tau^{-}\right)+\Delta p\left(k\tau\right)+\int_{k\tau}^{(k+1)\tau} P\left(p,q\right)\text{ }dt\text{ ,}$ (S. 9a)

$q\left((k+1)\tau^{-}\right)=q\left(k\tau^{-}\right)+\Delta q\left(k\tau\right)+\int_{k\tau}^{(k+1)\tau} Q\left(p,q\right)\text{ }dt\text{ ,}$ (S. 9b)

where $P\left(p,q\right)=p\left(1-p\right)\left(a_{2}-q\left(a_{1}+a_{2}\right)\right)$ and $Q\left(p,q\right)=q\left(1-q\right)\left(b_{2}-p\left(b_{1}+b_{2}\right)\right)$.

1. **Boundary stability**

First, consider the stability of the boundary $(0,0)$. If this vertex is to be stable, then it must be stable on the two edges $p=0$ and $q=0$ of the unit square. On the edge $q=0$, we have a simplified PD game among males. From the analysis of these games, we know that $p=0$ is stable if $a_{2}\tau+\ln\left(\left(1-\gamma_{1}\right)/\left(1-\gamma_{2}\right)\right)<0$ and unstable if this inequality is reversed. By a similar analysis of the simplified PD game among females on the edge $p=0$, we find $q=0$ is stable if $b_{2}\tau+\ln\left(\left(1-\psi_{1}\right)/\left(1-\psi_{2}\right)\right)<0$ and unstable if this inequality is reversed. Thus, stability of $\left(0,0\right)$ requires that $a_{2}\tau+\ln\left(\left(1-\gamma_{1}\right)/\left(1-\gamma_{2}\right)\right)<0$ and $b_{2}\tau+\ln\left(\left(1-\psi_{1}\right)/\left(1-\psi_{2}\right)\right)<0$.

Suppose that $a_{2}\tau+\ln\left(\left(1-\gamma_{1}\right)/\left(1-\gamma_{2}\right)\right)<0$ and $b_{2}\tau+\ln\left(\left(1-\psi_{1}\right)/\left(1-\psi_{2}\right)\right)<0$. Since $a_{2}\tau+\ln\left(\left(1-\gamma_{1}\right)/\left(1-\gamma_{2}\right)\right)<0$, on the edge $q=0$, $dp/dt=p\left(1-p\right)a_{2}>0$ and $p\left((k+1)\tau^{-}\right)<p\left(k\tau^{-}\right)$ when $0<p<1$ (since $\Delta p$ is sufficiently negative to more than reverse the increase in $p$ during the season). For $q>0$, $dp/dt<p\left(1-p\right)a_{2}$ and $\Delta p$ is the same as when $q=0$. Thus, the difference between $p\left((k+1)\tau^{-}\right)$ and $p\left(k\tau^{-}\right)$ is greater when $q>0$ than it is when $q=0$. In particular, $p\left((k+1)\tau^{-}\right)<p\left(k\tau^{-}\right)$ for all $0\leq q\leq1$. Thus, along any trajectory (that does not start on the edge $p=1$) we have $p\to0$. Once $p$ is sufficiently close to $p=0$, we know that $q\left((k+1)\tau^{-}\right)<q\left(k\tau^{-}\right)$ since $b_{2}\tau+\ln\left(\left(1-\psi_{1}\right)/\left(1-\psi_{2}\right)\right)<0$. Thus, $\left(0,0\right)$ is globally stable.

For convenience, define

$$\begin{matrix} \Gamma_{m}\equiv\frac{1}{\tau}\ln\left(\left(1-\gamma_{2}\right)/\left(1-\gamma_{1}\right)\right) \\ \Gamma_{f}\equiv\frac{1}{\tau}\ln\left(\left(1-\psi_{2}\right)/\left(1-\psi_{1}\right)\right)\text{ .} \end{matrix}$$

The above analysis yields the stability conditions in the first row of Table S2. The stability conditions in the other three rows follow from a similar argument showing that stability of the simplified PD games on the adjacent edges to a vertex implies global stability in the unit square. In particular, if one vertex is stable, it is globally stable.

**Table S2.**

| Boundary Equilibrium | Stability Conditions |
| --- | --- |
| $\left(0,0\right)$ | $a_{2}<\Gamma_{m}$ and $b_{2}<\Gamma_{f}$ |
| $\left(1,0\right)$ | $a_{2}>\Gamma_{m}$ and $b_{1}+\Gamma_{f}>0$ |
| $\left(0,1\right)$ | $a_{1}+\Gamma_{m}>0$ and $b_{2}>\Gamma_{f}$ |
| $\left(1,1\right)$ | $a_{1}+\Gamma_{m}<0$ and $b_{1}+\Gamma_{f}<0$ |

1. **Existence of interior $\tau$-periodic solutions**

In the rest of this section, suppose that no vertex is stable. For the existence of $\tau$-periodic solution, we have following theorem.

**Theorem 2.**  *If no vertex is stable in equation* **Eq.** **S.8***, then there is at least one $\tau$-periodic solution.*

**Proof.** Obviously, **Eq.** **S.8** has a $\tau$-periodic solution if and only if **Eq.** **S.9** has an interior equilibrium, which is the solution of equation

$F\left(p,q\right)\equiv\Delta p+\int_{k\tau}^{(k+1)\tau} P\left(p,q\right)\text{ }dt=0\text{ ,}$ (S. 10a)

$G\left(p,q\right)\equiv\Delta q+\int_{k\tau}^{(k+1)\tau} Q\left(p,q\right)\text{ }dt=0\text{ }.$ (S. 10b)

Without loss of generality, we assume that $a_{2}\tau+\ln\left(\left(1-\gamma_{1}\right)/\left(1-\gamma_{2}\right)\right)>0$. This implies $a_{1}\tau+\ln\left(\left(1-\gamma_{2}\right)/\left(1-\gamma_{1}\right)\right)>0$, $b_{1}\tau+\ln\left(\left(1-\psi_{2}\right)/\left(1-\psi_{1}\right)\right)<0$ and $b_{2}\tau+\ln\left(\left(1-\psi_{1}\right)/\left(1-\psi_{2}\right)\right)<0$ since no vertex is stable (see Table 2).

Then $F\left(p,0\right)>0$, $F\left(p,1\right)<0$, $G(p,0)=0$ and $G(p,1)=0$ for $0<p<1$. Also$G\left(0,q\right)<0$, $G\left(1,q\right)>0$, $F(0,q)=0$ and $F(1,q)=0$ for $0<q<1$. Consider the continuous-time dynamical system $dp/dt=F(p,q)$, $dq/dt=G(p,q)$ with continuous vector field given by $F(p,q)$ and $G(p,q)$. The edges of the unit square form a counterclockwise heteroclinic cycle for this dynamics. Since no interior trajectories converge to a point on one of these edges, the Poincare-Bendixson theorem [2] can be used to show that there is at least one interior equilibrium. Each such equilibrium corresponds to a $\tau$-periodic solution of **Eq.** **S.8**.

Unlike the corresponding result for the PD game with periodic impulses, the uniqueness of $\tau$-periodic solutions in the BS game with periodic impulses cannot be guaranteed in general. Numerical simulations suggest that there is only one interior $\tau$-periodic solution if $\tau$ is small, and that the interior $\tau$-periodic solution is not unique if $\tau$ is large. Generally, the number of $\tau$-periodic solutions depends on the relationship between $\tau$ and the periodic orbits of **Eq.** **S.8ab**.

Let $T_{\min}$ denote the shortest period of periodic orbit of **Eq.** **S.8ab**. In general, it is difficult to get the exact expression of $T_{\min}$, but simulation shows that it can be approximated by the period of interior periodic cycles near the interior equilibrium $(p^{*},q^{*})$. When the system state is near the interior equilibrium, **Eq.** **S.8ab** can be approximated by

$\frac{dp}{dt}=a\left(q^{*}-q\right)$ (S.11a)

$\frac{dq}{dt}=b\left(p^{*}-p\right)$ (S.11b)

where $a=p^{*}\left(1-p^{*}\right)\left(a_{1}+a_{2}\right)$ and $b=q^{*}\left(1-q^{*}\right)\left(b_{1}+b_{2}\right)$. By setting $y=q^{*}-q$ and $x=p^{*}-p$, **Eq.** **S.11** becomes

$\left(\begin{matrix} \frac{dx}{dt} \\ \frac{dy}{dt} \end{matrix}\right)=\left(\begin{matrix} 0 & -a \\ -b & 0 \end{matrix}\right)\left(\begin{matrix} x \\ y \end{matrix}\right)$ . (S.12)

The eigenvalues of the matrix $\left(\begin{matrix} 0 & -a \\ -b & 0 \end{matrix}\right)$ are $\lambda_{1},\lambda_{2}=\pm\text{i}\sqrt{-ab}$. Clearly, the solution of **Eq.** **S.12** is given by

$\left(\begin{matrix} x(t) \\ y(t) \end{matrix}\right)=\mathbf{l}\exp\left(\text{i}\sqrt{-ab}t\right)+\mathbf{m}\exp\left(-\text{i}\sqrt{-ab}t\right)$ , (S.13)

where **l** and **m** are eigenvectors corresponding to eigenvalues $\lambda_{1}$ and $\lambda_{2}$, and its period is $2\pi/\sqrt{-ab}$ which is the period of interior periodic cycles near the interior equilibrium $(p^{*},q^{*})$. Thus, $T_{\min}$ can be approximated as $2\pi/\sqrt{-p^{*}\left(1-p^{*}\right)\left(a_{1}+a_{2}\right)q^{*}(1-q^{*})(b_{1}+b_{2})}$.

From the numerical simulations, there appears to be a simple law for the maximum number of $\tau$-periodic solutions, denoted by $N$, which is given by $N=2\left[\tau/T_{\min}\right]+1$. Thus, if $\tau$ is less than $T_{\min}$, there is a unique interior$\tau$-periodic solution which is given by Theorem 2. Assume that $\tau$ is greater than $T_{\min}$ from now on and denote by $\Lambda\left(T\right)$ the periodic orbit of **Eq.** **S.8ab** with period $T$. Numerical simulations indicate that there are at most two $\tau$-periodic orbits between $\Lambda\left(\tau\right)$ and the boundary of the unit square (region III in Figure S1a); at most two $\tau$-periodic orbits between $\Lambda\left(\tau/\left(M-1\right)\right)$ and $\Lambda\left(\tau/M\right)$ for $M=2,3,\cdot\cdot\cdot,\left[\tau/T_{\min}\right]$ (region II in Figure S1a); and one in the region inside $\Lambda\left(\tau/M\right)$ for $M=\left[\tau/T_{\min}\right]$ (region I in Figure S1a). Figure S1 illustrates that this maximum number is achieved for specific impulsive parameters when payoff parameters are given by $a_{1}=3, a_{2}=2, b_{1}=-2, b_{2}=-3$ and $\left[\tau/T_{\min}\right]=2$ (i.e. there are exactly five $\tau$-periodic orbits).

Numerical simulations also indicate that the actual number of interior $\tau$-periodic solutions decreases as the impulsive effects increase (Figure S2). The payoffs for Figures S1 and S2 (i.e. $a_{1}=3, a_{2}=2, b_{1}=-2, b_{2}=-3$) are different than the payoffs used by Dawkins (see Figure 3 of the main text) since the three regions corresponding to Figure S1a for these latter payoffs are more difficult to discern. Nevertheless, our simulations using Dawkins’ payoffs show that there are also five interior $\tau$-periodic solutions for small impulsive effects and that this number decreases as the impulsive effects increase (Figure S2).

References:

1. Wang SC, Zhang BY, Li ZQ, Cressman R Tao Y (2008) Evolutionary game dynamics with impulsive effects. J Theor Biol 254: 384–389.

2. Hofbauer J, Sigmund K (1998) Evolutionary Games and Population Dynamics. Cambridge University Press, Cambridge.

**Figure Captions**

**Fig. S1.** **Five interior$\tau$-periodic solutions of the bimatrix replicator equation with periodic impulses of fixed intermediate strength for the BS game.** The equations $F\left(p,q\right)=0$ and $G\left(p,q\right)=0$ are represented by red and green curves, respectively, and the yellow curves represent $\Lambda\left(\tau/2\right)$ (the interior periodic cycles of the bimatrix replicator dynamic with period $\Lambda\left(\tau/2\right)$) (small cycle) and $\Lambda\left(\tau\right)$ (big cycle), where the parameters are taken as $a_{1}=3, a_{2}=2, b_{1}=-2, b_{2}=-3$, $\gamma_{1}=\psi_{1}=0$, $\gamma_{2}=\psi_{2}=0.1$, $T_{\min}\approx5.3$, $\tau=15$ and $\left[\tau/T_{\min}\right]=2$. **Fig. S1a** shows the three regions in the unit square formed by $\Lambda\left(\tau/2\right)$ and $\Lambda\left(\tau\right)$. From **Figs. S1b-d**, we can see that the solutions of $F\left(p,q\right)=0$ and $G\left(p,q\right)=0$ are isolated in three parts by $\Lambda\left(\tau/2\right)$ and $\Lambda\left(\tau\right)$. **Fig. S1d** shows that there are five $\tau$-periodic solutions (indicated by ⁭), where two are in region III, two are in region II, and one is in region I.

**Fig. S2. Interior$\tau$-periodic solutions of the bimatrix replicator equation with periodic impulses of varying intermediate strength for the BS game.** The equations $F\left(p,q\right)=0$ and $G\left(p,q\right)=0$ are represented by red and green curves, respectively, where the parameters are taken as $a_{1}=3, a_{2}=2, b_{1}=-2, b_{2}=-3$, $\gamma_{1}=\psi_{1}=0$, $T_{\min}\approx5.3$, $\tau=15$ and $\left[\tau/T_{\min}\right]=2$. In **Fig.S2a-c**, $\gamma_{2}$ and $\psi_{2}$ are 0.5, 0.9 and 0.99, respectively, and the average impulsive effects, $\Gamma_{m}$ and $\Gamma_{f}$, are -0.034, -0.11 and -0.23, respectively. From **Figs.S2a-c**, we can see that the number of $\tau$-periodic solutions decreases with the increase of impulsive effects.

**Figures**

**Fig.S1**


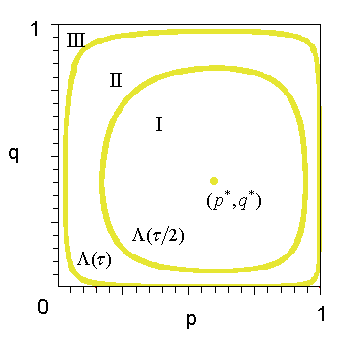
 ***
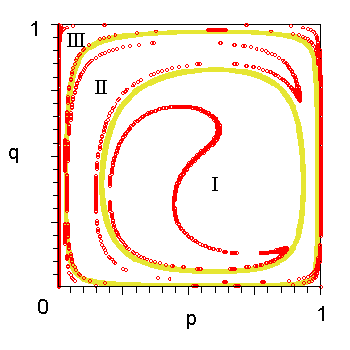
***

**Fig.S1a Fig. S1b**

***
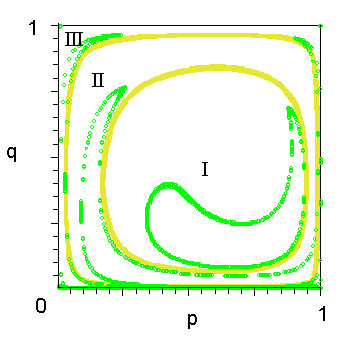

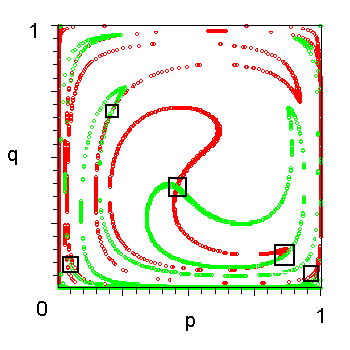
***

**Fig. S1c Fig. S1d**

**Fig.S2**

**
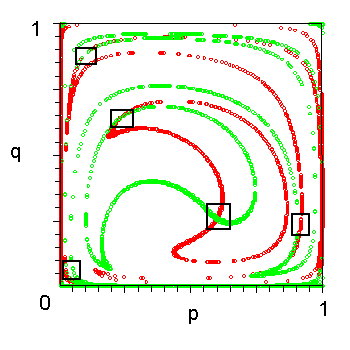

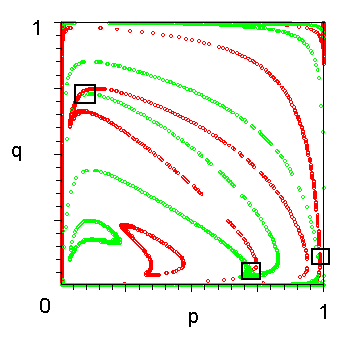

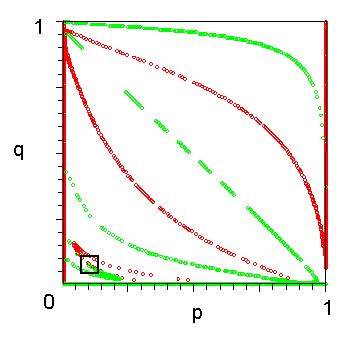
**

**Fig. S2a Fig. S2b Fig. S2c**
